# Monitoring and evaluating the adherence to a complementary food supplement (Ying Yang Bao) among young children in rural Qinghai, China: a mixed methods evaluation study

**DOI:** 10.7189/jogh.07.011101

**Published:** 2017-06

**Authors:** Qiong Wu, Yanfeng Zhang, Suying Chang, Wei Wang, Michelle Helena van Velthoven, Huijun Han, Min Xing, Li Chen, Xiaozhen Du, Robert W Scherpbier

**Affiliations:** 1 Department of Integrated Early Childhood Development, Capital Institute of Pediatrics, Beijing, China; 2 Health and Nutrition, Water, Environment and Sanitation Section, UNICEF China, Beijing, China; 3 Global eHealth Unit, Department of Primary Care and Public Health, Imperial College London, London, United Kingdom; 4 Department of Epidemiology and Biostatistics, Institute of Basic Medical Sciences, Chinese Academy of Medical Sciences, Peking Union Medical College, Beijing, China; 5 Department of Health Education in Framing and Pastoral Areas, Qinghai Health Education Center, Qinghai, China

## Abstract

**Background:**

Large investments are currently made in community–based complementary food supplement (Ying Yang Bao, YYB) programs to improve nutrition of young children in rural areas in China. However, there is a lack of knowledge about the experience and challenges of implementing YYB programs in China. We aimed to: 1) monitor distribution of YYB; 2) assess children’s adherence to and acceptability of YYB; and 3) evaluate community–based strategies to improve the program.

**Methods:**

This mixed methods evaluation study combined data from surveys and focus groups that took place during a controlled interventional evaluation trial. The trial aimed to evaluate the effectiveness of community–based YYB distribution on improving children's health status in rural areas in China. We conducted five cross–sectional surveys with caregivers of children aged 6–23 months (baseline survey (N = 1804) in August 2012 and four follow–up cross–sectional surveys: 1) N = 494 in January 2013; 2) N = 2187 in August 2013; 3) N = 504 in January 2014; and 4) N = 2186 in August 2014) in one rural county in Qinghai Province. We used a two–stage cluster sampling technique to select mothers with eligible children for each survey. Information was collected from caregivers on household characteristics, YYB consumption and acceptability in the surveys. High adherence in each survey was defined as children who consumed at least four YYB sachets during the previous week. A logistic regression model was developed to obtain odds ratios (OR) with 95% confidence intervals of factors associated with high adherence. Also, we conducted 10 focus groups with73 caregivers and health workers involved in the YYB distribution. Content analysis was used to explore qualitative findings, which were used to gain deeper insight into the quantitative results.

**Results:**

Around 90% of caregivers had ever received YYB and more than 80% of children ever took YYB. Caregivers mainly knew about YYB through their village doctors. High adherence to YYB increased from 49.4% in the first follow–up survey (January 2013) to 81.4% in the last follow–up survey (August 2014; *P* < 0.0001). Repeated training sessions with village doctors could increase adherence. However, due to unplanned YYB stock–out, caregivers did not receive YYB for six months, which may have led to a decrease of high adherence from 64.1% in the second follow–up survey (August 2013) to 53.6% in the third follow–up survey (January 2014; *P* < 0.0001). Self–reported acceptability increased from 43.2% to 71.8%, partly due to improving the taste of YYB, which was the main reason that children disliked taking YYB. Unfortunately, more than 60% of caregivers did not perceive positive health improvement in their children after taking YYB. Multivariate analysis showed that children with diarrhea (OR = 1.216, 95% CI 1.025–1.442), cough or fever (OR = 1.222, 95% CI 1.072–1.393) during the past two weeks had significantly lower adherence.

**Conclusions:**

This evaluation study showed that program monitoring in rural West China was critically important for understanding program implementation and adherence trends. This led to strategic changes to the intervention over time: improving the taste of YYB; strengthening health education of village doctors and caregivers; and ensuring continuity of YYB supply. Future programs need to monitor program implementation in other settings in China and elsewhere.

Although China has made great achievements in improving children's health during the past two decades, malnutrition of children is still a prominent problem, particularly in poor rural areas [1]. The prevalence of underweight and stunted Chinese children under–five was 8.0% and 20.3% in poor rural areas in 2010, respectively, which is more than two times as the national average. Furthermore, anemia prevalence of Chinese children aged 6–12 months and 13–24 months was 28.2% and 20.5% in 2010, respectively, and did not change between 2005 and 2009 [1]. Therefore, more efforts are still required to improve children’s nutrition and health in rural China.

During the last decade, multi–nutrient powders (MNPs), which are home nutrition fortification products, were developed and have been proposed as an important intervention for addressing undernutrition and micronutrient deficiencies among children younger than two years [2]. The efficacy of MNPs in reducing vitamin and mineral deficiencies and improving nutritional status of young children has been well documented in many countries [3-6]. In 2011, the World Health Organization (WHO) developed a guideline on how to use MNPs for home fortification of foods for children aged 6–23 months [7]. Also, MNPs programs are currently being scaled up at a national level in several developing countries, including Bangladesh [8], Mongolia [9], Kenya [5], Nepal [10] and Nigeria [11].

In China, a domestic produced MNP for infant and young children called Ying Yang Bao (YYB) was developed which contains essential fatty acids and protein through inclusion of full fat soy flour as well as multiple micronutrients [12,13]. Each sachet of YYB contained the following: protein (3.0 g), fat (1.0 g), carbohydrate (3.0 g), vitamin A (250 μg), vitamin D_3_ (5 μg), vitamin B_1_ (0.5 mg), vitamin B_2_ (0.5 mg), vitamin B_12_ (0.5 μg), folic acid (75 mg), elemental iron (7.5 mg), zinc (5 mg), and calcium (200mg). A small–scale efficacy study carried out in Gansu from 2001 to 2004 showed YYB can reduce anemia [14], and improve children’s developmental quotient (DQ) [15]. With this evidence on the efficacy of YYB, the Chinese government approved and issued the National Standard for Complementary Food Supplements (GB/T22570–2008) [16] in 2009 and made YYB commercially available on the market [12]. Moreover, YYB was recommended for scale–up in disaster and poor rural areas to improve Chinese children’s health. Between 2010 and 2011, free YYB was provided to around 30 000 children aged 6–23 months in eight earthquake–affected counties in Sichuan, Gansu and Shaanxi provinces, supported by the United Nations Children’s Fund (UNICEF), the United States Centers for Disease Control (US CDC) and China CDC [13] In 2011, the Chinese National Health and Family Planning Commission and All–China Women's Federation initiated a national community–based nutritional program to improve children's nutrition in poor rural areas, which provides free YYB for children aged 6–23 months in poor rural areas [17]. This program was scaled up between 2013 and 2014 [18–20] and until 2014 the program had covered 341 counties in 21 provinces in China, which was estimated to reach more than one million children aged 6–23 months in rural areas [20].

In addition, as a part of Qinghai–Tibet Plateau, the provincial government of Qinghai has also been providing free YYB to all children aged 6–23 months in 15 out of 34 poor counties in Qinghai Province since 2012, which were consistent with the national program [21]. We carried out a controlled interventional evaluation trial in Qinghai from 2012 to 2014 to evaluate the effectiveness of community–based YYB distribution on improving children's health status in rural areas in China, and reported that community–based complementary food supplements combined with dietary counseling can improve feeding practices and reduce anemia prevalence [22].

Although such a large–scale national nutritional program was carried out in China, no study documented the adherence, program experience and challenges of program with the community–based distribution approach. High adherence to MNPs is critical for achieving the maximum health benefits of the intervention. Based on the controlled interventional trial in Qinghai, this current paper aimed to: 1) monitor distribution of YYB; 2) assess children's adherence to and acceptability of YYB; and 3) evaluate community–based strategies to improve adherence. This will illustrate how monitoring led to strategic changes in the intervention that might be helpful for improvement of larger scale programs in China and elsewhere.

## METHODS

### Study design and data sources

This current mixed methods evaluation was embedded in the controlled interventional trial in Qinghai [22]. We combined data from surveys and focus groups.

Caregivers and their children aged between 6–23 months were main participants of our evaluation. Monitoring of YYB distribution and evaluation of children’s adherence to and acceptability of YYB only took place in the intervention county in the trial, and therefore the data in this current paper are from the intervention county (**Table 1**). Quantitative data were from five representative cross–sectional surveys, which aimed to assess coverage of YYB distribution, children’s adherence to YYB, caregivers’ experience with YYB, YYB awareness and lessons learnt from the YYB program. Qualitative data were from ten focus group discussions with local health workers and caregivers to increase our understanding of program implementation. We integrated quantitative and qualitative data to show findings on intervention implementation. Also we compared the qualitative findings with quantitative data to validate the quantitative findings [23]. We first report quantitative data followed by qualitative data for YYB distribution, adherence of children to YYB, caregivers’ experience with YYB, YYB awareness and lessons learnt from the YYB program. In addition, we report qualitative data only on difficulties with YYB distribution.

**Table 1:** Sources of monitoring data for consumed complementary food supplement Ying Yang Bao (YYB) intervention

Data source	Participants	Type of research	Date	Number of months after intervention
Baseline survey	Children aged 6–23 months and their caregivers (n = 1804)	Quantitative	August 2012	
Intervention started			September 2012	
Six focus groups	MCH workers in township hospitals (n = 11); village doctors (n = 20); fathers (n = 6), mothers(n = 4), and grandparents (n = 9) of children aged 6–23 months	Qualitative	November 2012	2 months
Mini 1 survey	Children aged 6–23 months and their caregivers (n = 494)	Quantitative	January 2013	4 months
Four focus groups	Mothers (n = 12) and grandparents (n = 11) of children aged 6–23 months	Qualitative	April 2013	7 months
Midterm survey	Children aged 6–23 months and their caregivers (n = 2187)	Quantitative	August 2013	11 months
Mini 2 survey	Children aged 6–23 months and their caregivers (n = 504)	Quantitative	January 2014	16 months
Endline survey	Children aged 6–23 months and their caregivers (n = 2186)	Quantitative	August 2014	23 months
Intervention ended			August 2014	

### Study setting

The provincial government had already decided to implement the program in 15 counties in Qinghai Province before we designed the trial and therefore we selected one intervention county, Huzhu County, from these counties. We selected the control county, Guinan County, from the remaining 19 counties in Qinghai. For selection, we considered the willingness of the local government to cooperate, and socio–economic conditions between the two counties, including: annual per capita income for rural residents, the adult female literacy rate, and the proportion of piped water coverage.

The intervention county lies in the northeast of Qinghai province, with the area of 3423.9 km^2^. It has total population of 370 540, with 93.1% of rural population. There are 19 townships and 294 villages in the intervention county. The annual per capita income of rural residents is ¥ 5691 (US$ 872.43) in 2011 [24].

### Quantitative approach

We conducted a baseline survey in August 2012 and four follow–up cross–sectional surveys in January 2013 (mini 1 survey), August 2013 (midterm survey), January 2014 (mini 2 survey), and August 2013 (endline survey), respectively, in the intervention county (**Table 1**). Main caregivers and their children aged between 6–23 months were participants of our evaluation.

### Sample size and sampling

The sample size and two–stage sampling procedure were reported in the effectiveness of the controlled interventional study paper [22]. We used a sample size of 1973 in the baseline survey, midterm survey and endline survey, as the data on weight, height and hemoglobin level were collected. However, in the mini 1 and mini 2 surveys, we only collected the data on hemoglobin levels, and thus we used a sample size of 504 in both surveys.

We conducted the surveys in the same villages; this meant that children could be included in more than one survey.

### Data collection

We used the adapted Maternal, Newborn and Child Health household survey (MNCH HHS) tool [25] to collect baseline characteristics and follow–up data, which included socio–demographic characteristics, infant and young child feeding, and morbidity status. Trained fieldworkers from the School of Public Health, Qinghai University collected data for the five surveys using smartphones. During each survey, we asked caregivers to first come to village clinics for registration, and then interviewers conducted interviews with caregivers in village clinics.

For the four follow–up surveys, we developed questions on YYB distribution and consumption using information that we obtained from a pilot text messaging survey in October 2012 (see Appendix S1 in **Online Supplementary Document(Online Supplementary Document)**).

### Definition of high adherence

The outcome variable adherence was measured through a question in the questionnaire “How many sachets of YYB did your child consumed during the previous week?” High adherence was defined as the proportion of children who consumed at least four YYB sachets during the previous week, which consist with the definition in other studies [6].

### Definition of YYB acceptability

Children’s acceptability was measured though one question in the questionnaire “How do you think your child like taking YYB? 1=Like very much; 2=Liked; 3=Neutral; 4=Disliked at the beginning, but liked after a while; 5=Disliked, reasons for dislike….; 8=Don’t know.”

### Statistical analysis

Data of each interview was automatically stored as “.txt” form in each smartphone, and we manually transformed and pool each data into a Microsoft Excel (Microsoft, Seattle, Washington, WA, USA) sheet for each survey. After the data cleaning, we converted the database into databasefile (dbf) for the final analysis. We carried out statistical analysis with SAS 9.2 for Windows (SAS Institute Inc., North Carolina, USA). We present the mean and standard deviation (SD) to describe the age of mothers and main caregivers of children, and mean sachets of YYB consumed by children surveyed during the previous week in each survey. We used ANOVA [26] analysis to detect statistically significant differences in age, and T–test to compare differences for the mean sachets. For binary or categorical variables, we present percentages. We used Pearson χ^2^–test and Fisher exact test to compare binary and categorical variables. The denominators were all the participants in each survey, including those who answered “Don’t/Didn’t know” in several questions.

We used logistic regression to identify factors associated with high adherence to YYB. We combined the data from the four follow–up surveys to explore the factors. All relevant factors were first selected by single factor analysis. Multivariate analysis was used to assessed, and only those that were significant included in the final multivariate model are presented. Models were adjusted for the relevant covariates using stepwise regression. We present Odds Ratios (OR) and 95% confidence intervals (CI). We considered two–tailed *P*–values of <0.05 for a significant difference.

### Qualitative approach

We conducted 10 focus group discussions in the intervention county to obtain a better understanding of YYB implementation: six in November 2012 and four in April 2013 (**Table 1**).

### Sampling

We used convenience sampling. The participants in the focus group were independent from the surveys. .MCH workers came from different township hospitals in the county (1 focus group), village doctors were from different villages in a township (2 focus groups). Caregivers were from the same villages and had a child aged 6–23 months (7 focus groups).

### Data collection

One local facilitator from Qinghai Health Education Center and one researcher from Capital Institute of Pediatrics conducted focus group discussions. The study team developed the focus group guides (Appendix S1 in **Online Supplementary Document(Online Supplementary Document)**). Discussion with MCH workers and village doctors were done at a place convenient for them. Caregivers were invited to village clinics to participate. Discussions were conducted in Mandarin, typically lasting around 30 minutes, and were digitally recorded with the permission of each participant. Tape recordings were transcribed verbatim in Chinese by a medical student from Qinghai University, and then checked by another medical student by listening to the tapes again to correct any errors. Finally, the study team member who participated in the focus groups validated the transcripts.

### Analysis

We conducted content analysis [27] by examining the major themes and patterns that emerged from the data. Two Chinese researchers involved in the study (WQ and DXZ) first read the transcripts and use MAXQDA 11 (VERBI Software GmbH, Berlin, Germany) to organize data along the previously identified key themes independently. Then the researchers compared the themes and discussed areas of agreement and discrepancies. They further refined the themes until consensus was reached on the themes and interpretation of the findings. Finally, WQ translated the themes and related quotes into English and DXZ reviewed the translated themes. We list all the key themes that we identified.

### Ethical considerations

The evaluation study was approved by the Ethical Committee of Capital Institute of Pediatrics. All interviewees read the information sheet and provided written informed consent.

## RESULTS

### Population in quantitative surveys

All caregivers who were invited agreed to participate in the cross–sectional surveys (**Table 2**). Around 30% of children were currently breastfed. Two–week prevalence of cough/fever, and diarrhea were around 40% and 15%, respectively. In all five surveys, more than half of the main caregivers were mothers and around 30–40% were grandparents. The mean age of main caregivers was 40 years and around 40% of them were illiterate.

**Table 2:** Characteristics of surveyed caregivers and their children

Surveys	Baseline (N = 1804)	Mini 1 (N = 494)	Midterm (N = 2187)	Mini 2 (N = 504)	Endline (N = 2186)
**Children**					
Age, % (n)					
6–11 months	33.8 (610)	29.6 (146)	39.6 (866)	25.6 (144)	35.5 (775)
12–17 months	26.8 (484)	41.5 (205)	29.5 (645)	37.7 (190)	29.1 (635)
18–23 months	39.4 (710)	28.9 (143)	30.9 (676)	33.7 (170)	35.5 (776)
Sex, % (n)					
Boy	53.2 (960)	54.3 (268)	55.0 (1203)	58.5 (295)	54.8 (1198)
Girl	46.8 (844)	45.7 (226)	45.0 (984)	41.5 (209)	45.2 (988)
Currently breastfeeding	26.8 (484)	36.2 (179)	27.1 (593)	27.8 (140)	25.3 (553)
Two–week prevalence of cough or fever	49.0 (884)	38.7 (191)	35.3 (772)	43.9 (221)	39.8 (870)
Two–week prevalence of diarrhea	16.7 (302)	17.6 (87)	14.8 (324)	14.5 (73)	15.9 (348)
**Mothers**					
Age (year), mean (SD)	26.9 (4.9)	27.4 (4.6)	29.1 (11.1)	29.2 (10.4)	28.6 (9.6)
Mother working outside hometown	24.1 (435)	11.9 (59)	26.1 (569)	12.3 (62)	13.6 (515)
Father working outside hometown	39.2 (707)	47.6 (235)	63.9 (1397)	41.1 (207)	57.5 (1257)
**Main caregivers**					
Relationship with children, % (n)					
Mother	53.2 (960)	64.8 (320)	52.4 (1146)*	58.5 (295)	51.8(1131)†
Father	0.6 (11)	0.8 (4)	0.4 (8)*	3.2 (16)	0.2 (4)†
Grandparent	45.0 (812)	34.4 (170)	46.7 (1020)*	34.3 (173)	47.8(1045)†
Other	1.2 (21)	0.0 (0)	0.5 (12)*	4.0 (20)	0.2(5)†
**Age (year), mean (SD)**	39.4 (13.7)	36.3(12.3)	40.0 (13.6)*	38.3 (13.2)	38.8(14.1)
Education,% (n)					
Illiterate	41.3(745)	33.4 (165)	40.2 (879)‡	40.1 (202)	40.3(880)†
Primary school	22.0 (396)	23.7 (117)	21.4 (467)‡	17.3 (87)	18.9(414)†
Junior high school	32.0(578)	35.0 (173)	31.5 (689)‡	37.9 (191)	34.1(746)†
Senior high school or above	4.1 (74)	7.3 (36)	5.7 (125)‡	4.6 (23)	5.9(128)†
Do not know	0.6 (11)	0.6 (3)	1.1 (25)‡	0.2 (1)	0.8 (17) †

*One interviewee was missing for this calculation.

†Two interviewees were missing for this calculation.

‡Two interviewees were missing for this calculation.

### Population in focus groups

A total of 73 people participated in the focus groups: 11 township MCH workers, 20 village doctors, 6 fathers, 16 mothers, and 20 grandparents.

### YYB distribution

We found in the four follow–up cross–sectional surveys that most caregivers (around 90%) of children aged 6–23 months in villages had ever received YYB (**Table 3**).Both health workers and caregivers in focus groups said that YYB was mainly distributed by village doctors from September 2012 (after the baseline survey). Once a month, village doctors received YYB from their township hospitals, and then distributed to caregivers through home visits or by asking caregivers to visit clinics through mobile phone calls. Furthermore, YYB was given to children who received vaccinations in the clinics. In some villages, caregivers had to use empty YYB bags and boxes to exchange a new box of YYB to ensure children consumed YYB they had received. Due to different number of children in different villages, it took village doctors one to seven days to distribute YYB for one round. Every month MCH workers went to their catchment villages to monitor YYB distribution while they were undertaking their regular supervision of the basic public health service program.

**Table 3:** Complementary food supplement Ying Yang Bao (YYB) distribution and consumption by children

	Mini 1 (N = 494) (4 months)	Midterm (N = 2186*) (11 months)	Mini 2 (N = 496)† (16 months)	Endline (N = 2186) (23 months)		P1‡	P2§	P3‖
Proportion of children whose caregivers ever received YYB	87.7% (433)	97.1% (2123)	95.6% (474)	99.0% (2164)	<0.0001		0.0746	<0.0001
Proportion of children who ever consumed YYB	82.0% (405)	95.9% (2096)	93.6% (464)	98.1% (2144)	<0.0001		0.0243	<0.0001
Proportion of children who were currently still consuming YYB¶	–	82.3% (1800)	73.1% (363)	92.9% (2032)	–		–	–
Proportion of children who took YYB within the last 24 hours	23.5% (116)	3.3% (1383)	48.2% (239)	78.8% (1722)	<0.0001		<0.0001	<0.0001
Mean (standard deviation) sachets of YYB consumed by children surveyed during the previous week	4.0 (3.0)	4.7 (2.9)	3.9 (3.1)	5.8 (2.2)	0.0002		<0.0001	<0.0001
Proportion of children who had high adherence (consumed 4 sachets of YYB or more)	49.4 (244)	64.1 (1402)	53.6 (266)	81.4 (1780)	<0.0001		0.1824	<0.0001

*Data missing for 1 child.

†Data missing for 8 children.

‡Mini 1 vs Midterm.

§Mini 1 vs Mini 2.

‖Mini 1 vs Endline.

¶We did not ask caregivers this question in the Mini 1 survey.

Although most caregivers of children had ever received YYB, still a small part of caregivers did not receive YYB. **Table 4** shows the distribution of reasons why caregivers did not receive YYB and we found in main reasons for “not received” were “caregivers didn’t know the distribution of YYB”, and “children were just six months” in four follow–up surveys.

**Table 4:** Reasons for “not received complementary complementary food supplement Ying Yang Bao (YYB)”

Reasons	Mini 1	Midterm	Mini 2	Endline
Didn't know the distribution of YYB	28	13	5	4
Children were just six months	12	15	6	5
Not at home when distribution	1	7	0	3
Didn't want YYB	2	0	1	1
There is no YYB in the village clinic	0	2	3	3
Others	4	6	2	1
Didn't know	5	14	25	5

### Adherence of children to YYB

In general, the coverage of YYB consumed by children increased with the progress of the YYB program implementation (**Table 3**). The proportion of children who took YYB within the last 24 hours increased from 23.5% in the mini 1 survey to 78.8% in the endline survey (*P* < 0.0001); meanwhile, the average sachets children consumed during the previous week increased from 4.0 sachets to 5.8 sachets (*P* < 0.0001). The proportion of children who had high YYB adherence (took at least four YYB sachets during the previous week) for each follow–up cross–sectional survey was 49.4%, 64.1%, 53.6% and 81.4%, respectively, which indicated a similar trend. However, there were a significant decrease in all adherence–related indicators between the midterm and mini 2 surveys (*P* < 0.0001) (**Table 3**).

**Figure 1:**
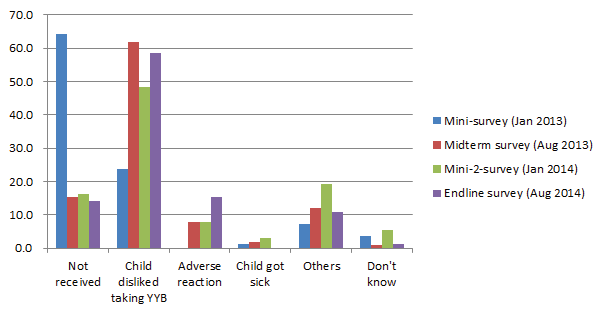
Reasons children had never consumed complementary food supplement Ying Yang Bao (YYB). The denominator of this figure were the numbers of caregivers whose children had never received or consumed YYB or stopped consuming YYB currently, with 89, 386, 133, 154 in each follow–up survey, respectively.

In the first mini survey, the main reason why children had never consumed or stopped consuming YYB currently was “Not received”; however, in the last three follow–ups, the main reason changed to “children disliked taking YYB.” (**Figure 1**).

In multivariate analysis, higher age of the children (OR = 0.976, 95% CI 0.962–0.990), father working outside hometown (OR = 0.795, 95% CI 0.692–0.913) were significantly associated with having high adherence (**Table 5**). However, had diarrhea (OR = 1.216, 95% CI 1.025–1.442), had cough or fever (OR = 1.222, 95% CI 1.072–1.393) during the past two weeks were associated with having low adherence.

**Table 5:** Factors associated with high adherence to complementary food supplement Ying Yang Bao (YYB)

Factors	β	Wald	*P*–value	OR (95% CI)
Age of child (months)	–0.0244	10.5029	0.0012	0.976 (0.962, 0.990)
Main caregiver:				
Mother – grandparents	0.1271	0.7757	0.3785	1.136 (0.856, 1.507)
Mother – father	0.4695	1.0964	0.2951	1.599 (0.664, 3.851)
Mother – others	0.0063	0.0001	0.9904	1.006 (0.769, 1.030)
Age of main caregiver (years)	–0.0063	1.6150	0.2038	0.994 (0.984, 1.003)
Main caregiver attend middle school or above	0.1188	2.3153	0.1281	1.126 (0.966, 1.312)
Mother working outside hometown	–0.1161	1.2767	0.2585	0.890 (0.728, 1.089)
Father working outside hometown	–0.2293	10.5547	0.0012	0.795 (0.692, 0.913)
Main income source of family was work	–0.1163	2.4428	0.1181	0.890 (0.769, 1.030)
Child was currently breastfed	0.0982	1.2669	0.2603	1.103 (0.930, 1.309)
Diarrhea	0.1958	5.0589	0.0245	1.216 (1.025, 1.442)
Cough and fever	0.2005	9.0021	0.0027	1.222 (1.072, 1.393)
Surveys:				
Midterm – Mini 1	–0.4450	16.6102	<0.0001	0.641 (0.517, 0.794)
Mini 2 – Mini 1	–0.0771	0.3110	0.5770	0.926 (0.706, 1.214)
Endline – Mini 1	–1.3838	148.7340	<0.0001	0.251 (0.201, 0.313)

OR – odds ratio, CI – confidence interval

**Figure 2:**
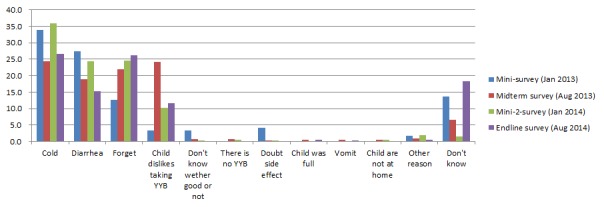
Situations where caregivers temporarily skip sachets of consumed complementary food supplement Ying Yang Bao (YYB) to their children. The denominator of this figure is the numbers of caregivers whose children are still consuming YYB.

Moreover, caregivers also reported that the most common situation in which they temporarily skipped sachets of YYB to their children was because children got cold or diarrhea (**Figure 2**). The proportions of children who temporarily skipped sachets of YYB due to sickness were higher in the mini 1 and mini 2 surveys which were undertaken in January, compared to the midterm and endline surveys which were undertaken in August. In addition, the proportion of caregivers who skipped sachets of YYB to their children because of forgetting increased throughout the four follow–up surveys.

“Not feed (YYB) when my child got a cold, (I am) afraid that YYB could not be given with medication for a cold together.” (a grandparent, focus group in April 2014)

### YYB acceptability

**Figure 3:**
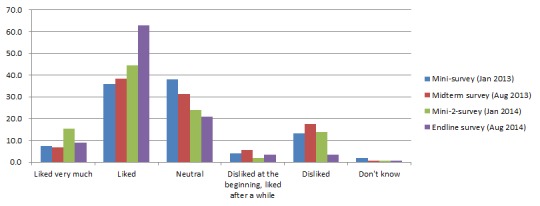
Caregivers’ perception of child acceptance of consumed complementary food supplement Ying Yang Bao (YYB) in the follow–up surveys. Caregivers’ experience with YYB.

**Figure 3** shows that most children’s perceptions on YYB were either “neutral” or “like” YYB. The proportion of children who liked taking YYB increased over time; at the time of the endline survey more than 70% caregivers reported that their children liked taking YYB. The most common reason for disliking YYB reported by caregivers were that children disliked the taste of YYB 50.9% (27/53) in the Mini 1 survey, 57.2% (179/313) in the midterm survey, 86.0% (43/50) in the Mini 2 survey, and 67.6% (48/71) in the endline survey). Other reasons were: “did not know why children disliked taking YYB”, “nausea and vomit”, and “diarrhea”.

In all focus groups, MCH health workers, village doctors and caregivers said that the taste of YYB should be improved. Some caregivers said that their children did not like taking YYB just to the soybean taste and smell. They suggested that it should be changed into a sweeter taste that children like, by for example adding some sugar.

*“The critical problem is that the taste of YYB is not good, and children are not willing to consume. (YYB) tasted like soybean milk powder. Children even refused eating meals, when YYB added to their meals.”* (a village doctor, focus group in November 2013)

In all follow–up surveys, more than 60% of caregivers did not perceive any change in their children after they started giving YYB to their children (**Table 6**). However, only less than 20% of caregivers had perceived positive weight gains and diseases prevented in their children; less than 10% of caregivers perceived positive height gains, increased appetite and improved children’s cognitive ability.

**Table 6:** Mothers’ experience with complementary food supplement Ying Yang Bao (YYB)*

Variables (N = 78)	Mini 1 (N = 405) % (n)	Midterm (N = 1800) % (n)	Mini 2 (N = 363) % (n)	Endline (N = 2030) % (n)	*P*1†	*P*2‡	*P*3§
No changes observed	63.5 (257)	61.7 (1110)	64.2 (233)	74.7 (1517)	0.5025	0.8334	<0.0001
Perceived changes in child’s health after feeding YYB:							
Positive weight gains	19.0 (77)	18.4 (331)	12.1 (44)	12.1 (246)	0.7703	0.0089	0.0002
Positive height gains	7.2 (29)	7.2 (130)	3.9 (14)	5.2 (106)	0.9654	0.0585	0.1195
Increased appetite	9.6 (39)	8.2 (147)	8.8 (32)	7.7 (156)	0.3385	0.6973	0.1879
Prevented diseases	13.1 (53)	16.7 (300)	8.3 (30)	12.0 (244)	0.0758	0.0316	0.5492
Increased cognitive ability	0.5 (2)	3.6 (64)	1.9 (7)	3.3 (66)	0.0011	0.0651	0.0021

*The denominator of this table were the numbers of caregivers whose children were still consumed YYB currently.

†Mini 1 vs Midterm.

‡Mini 1 vs Mini 2.

§Mini 1 vs Endline.

Several caregivers in focus groups mentioned that the appetite, growth, immunity of their children had improved and less colds occurred after eating YYB; however, some caregivers said it was too short to see any changes in their children.

*“My child is heavier than before, and he has never got cold, even if I took him out every day. Now he is nine months, but he is able to walk by holding something, and grasp things himself.”* (a grandfather, focus group in November 2013)

### YYB awareness

**Figure 4:**
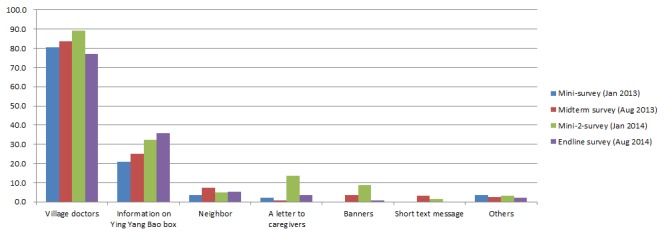
Source of caregivers' information on consumed complementary food supplement Ying Yang Bao (YYB).

In the follow–up surveys, the proportion of caregivers who reported that they had ever received the information on YYB increased from 43.5% in the mini–1 survey to 64.1% in the endline survey. Village doctors were the first source of YYB information and caregivers who received YYB information from village doctor were around 80% in each survey (**Figure 4**). The other major source of YYB information was YYB box, and the proportion of caregivers reporting that they ever received YYB information from the box increased throughout the four follow–up surveys.

Qualitative data showed that village doctors played very important roles in YYB health education. *“Village doctor told me that YYB can provide calcium, iron, zinc and vitamins to children” *(a grandfather, focus group in November 2013).

When distributing YYB to caregivers, village doctors encouraged them to give YYB to their children. Some village doctors demonstrated in their clinics to caregivers how to give YYB to children.

“*When I came to the village clinic to bring YYB, the village doctor told me the benefit of YYB. After I came back home, I read from the introduction book [on the YYB box].”* (a mother, focus group in November 2013)

### Difficulties with YYB distribution

MCH workers from township hospitals in the intervention county generally agreed that more than half of the village doctors in their townships had a positive attitude toward YYB distribution; however, some village doctors also made complaints.

“We are very busy, and still have to distribute YYB.”

*“Although we work very hard on YYB distribution, caregivers are ungrateful.”* (a village doctor, focus group in November 2013)

Village doctors indicated that several caregivers in the villages were uncooperative and reluctant to feed their children YYB or some children disliked taking YYB.

“*There is a grandmother in my village who did not feed YYB to her grandchild. I called her for three times to encourage her to feed, but she still told me her child disliked taking YYB.*”

In addition, there was no allowance on YYB distribution for health workers. Several village doctors said that the YYB distribution increased their work load. Every month, they had to use two to four working days to inform of caregivers and distribute YYB. Sometimes they had to bring YYB door to door if caregivers did not come to village clinics. Village doctors had to pay all the fees for calls and transportation during the YYB distribution. Both MCH workers and village doctors asked whether village doctors could be given allowance.

### Lessons learnt from YYB program implementation

We initially developed a leaflet called “a letter to caregivers”, which contained a detailed description of YYB benefit and usage. However, at the baseline survey (August 2012) we found that more than 40% of main caregivers were illiterate. Therefore, we encouraged village doctors to deliver YYB information through face–to–face counseling and we modified the leaflet by adding more pictures to replace the text description. However, only less than 10% of caregivers reported that they got information of YYB from the leaflets in four follow–up surveys (**Figure 4**).

After the first two months of program implementation, we carried out focus groups with MCH health workers, village doctors, and caregivers of children. The following problems were identified: children disliked the taste of YYB; children refused to take YYB; stopped giving YYB when their children got sick; and side effects, such as diarrhea after children took YYB.

In the first mini survey (4 months after intervention start, January 2013), we found that 61.3% of caregivers temporarily skipped sachets of YYB to their children when they were sick (cold or diarrhea) (**Figure 2**), and 17.0% of mothers reported that their children disliked consuming YYB (**Figure 3**). Also, 12.6% of caregivers sometimes forgot to give YYB (**Figure 2**) and 12.3% of caregivers did not receive YYB (**Table 3**). Therefore, additional training was conducted in March 2013 to train the village doctors in Qinghai YYB project counties to help them with dealing with those problems. Moreover, we used leaflets, banners, calendars and posters to promote the program.

In the midterm survey (11 months after intervention start, August 2013), no new program problems were identified (August 2013).However, caregivers who forgot to give YYB and children who disliked YYB significantly increased to 22.0% (*P* < 0.001, **Figure 2**)and 23.1% (*P* = 0.008, **Figure 3**), respectively . Therefore, we continued encouraging village doctors to explain again the benefit of YYB, with a focus on caregivers who forgot to give YYB and children who disliked YYB. Meanwhile, banners, calendars and posters were still be used.

In September 2013, there was a stock–out of YYB without further provision from the provincial health department, because a new lengthy approval procedure for YYB procurement was in process. As YYB is a governmental program, purchasing YYB is regulated under a complex and strict process after every 12 months of intervention implementation to ensure the good quality and reasonable pricing. As a result, YYB provision had to be stopped from September to October 2013. We frequently communicated with the county MCH hospital in the intervention county to monitor the process of implementation. To make sure the program was continuously implemented, UNICEF decided to provide YYB to the intervention county for two months from November to December 2013. However, in January and February 2014 YYB was still unavailable, because the approval procedure had not been completed yet.

In the Mini–2 survey (16 months after intervention start, January 2014), the percentage of children who took YYB within the last 24 hours and high adherence significantly decreased as a result of the YYB stock–out (**Table 3**). We coordinated with provincial health department to speed up the approval procedure. Also we requested the manufacturer to improve the taste of YYB.

After governmental approval, the YYB supplier changed from “Tian Tian Ai (天添爱)” to “Fu Ge Sen (福格森)” and YYB was re–supplied from March 2014 onward. In June 2014, we carried out a three–day training sessions to retrain all the village doctors in the intervention county to strengthen their YYB related knowledge, health education and complementary feeding skills. Most village doctors in the training sessions said that the taste of the new YYB was much better than before and that children in their village liked YYB more which was reflected by a decrease in the proportion of children who disliked YYB at the time of the endline survey in August 2014 (23 months later survey) (**Figure 3**). Also at the endline survey high adherence to YYB increased significantly (**Table 3**). However, because the proportion of caregivers who forgot to give YYB continuously increased (**Figure 2**), we advised the local MCH hospital to continue using multiple channels to promote caregivers’ awareness of the program.

## DISCUSSION

Currently, the Chinese government invests more than￥500 million RMB (US$ 75.24 million) yearly to implement the community–based complementary food supplement program (YYB program) to improve children’s health in rural counties in China since 2014 [18]. However, there was no data published on YYB program implementation experiences and challenges in China. Although our study was only carried out in one Chinese rural county, it provides an important insight into successfully implementing a community–based complementary food supplement program in China. The coverage of YYB distribution was high; the majority of caregivers ever received YYB and most children ever took YYB, which indicated that YYB was efficiently delivered to caregivers in the program county by the multi–tiered distribution channel. A previous study in earthquake–affected areas in China also proved the distribution system from manufacturer to MCH hospitals to township clinics, then to village doctors could guarantee the receipt of YYB [13]. Generally, children’s adherence to YYB increased over time in our study, and the proportion of high adherence got to 81.4% at the endline survey. Caregivers reported children’s acceptability to YYB increased over time as well, and the main reason for dislike was the taste of YYB. Therefore, we requested the YYB manufacturer to improve the taste of YYB, which appeared to result in children liking YYB better and taking more doses. Unfortunately, more than 60% of caregivers did not perceive positive health improvement in their children after taking YYB. More than 60% of caregivers who ever received YYB were given information on YYB and the main information sources were village doctors and YYB boxes.

Program monitoring is critical for understanding program implementation and enabling more strategic implementation [28,29]. In our study, we carried out both quantitative and quantitative interviews to monitor the program for two years, which provided us dynamic and comprehensive information on program implementation and allowed us to make real–time modifications. For example, when we found more than 40% of main caregivers were illiteracy at the baseline survey, we changed the text information on the leaflet into the pictures, which was easy for illiterate caregivers to understand. Although we also used leaflets, calendar, banners, posters and blackboards to promote the program, most caregivers reported they received information on YYB from village doctors in each survey, which indicated that well–trained village doctors played an important roles in successful program implementation. Therefore, distribution of YYB as well as health education relied mainly on village doctors. We repeatedly undertook quality training sessions for raising awareness and educating village doctors and caregivers [29]. When monitoring data showed that overall adherence to YYB was low, we conducted additional training sessions with village doctors (March 2013 and June 2014), which helped increase the adherence to YYB. Data in the midterm and endline surveys showed that the high adherence to YYB increased after the training.

Interventions like MNP that aim to reduce anemia prevalence in rural communities will only work when high levels of acceptance and adherence are reached [6,30]. Previous studies in other countries showed high adherence (defined as consumption of four sachets or more per week) to daily provision of MNP ranged from 32% to around 90% [6]. Studies of MNP in Bangladesh even found an adherence of 70–100% [30–33]. The highest adherence to MNP in those studies was observed in a study that was conducted in a controlled setting where field workers deliver and monitor the intervention on a regular basis [31]. A study providing daily sprinkles micronutrient powders to children for 2 months had an average of 75% adherence, but only 39% of children took all 60 sachets [34]. Studies found that a longer duration of the intervention decreased people’s motivations and adherence [31,35]. Data in our study showed that the high adherence increased from 49.4% in the mini1 survey (January 2013) to more than 80% in the endline survey (August 2014), which indicates that when active program monitoring to address challenges, adherence can increase over time.

We found in our study that children who got cough/fever or diarrhea during the past two weeks had significant lower adherence, which consist with most caregivers’ report that they would skip sachets of YYB when their children got cold or diarrhea. Mirak et al. also found in Bangladesh that around 19% of the mothers reported that they skipped a sachet of MMNP because of any children’s illness in the past 60 days, and nearly half of those who skipped a sachet of YYB had fever in the past 15 days [30].

In addition, our study suggested that one of the main reasons for poor acceptability and adherence was that children disliked or even refused to eat YYB, which implied that the taste of YYB needed to be improved. A study in Lao People’s Democratic Republic also report sprinkles had unpleasant smell and taste [4]. Different to MNP in other counties, Chinese YYB was a full fat soybean powder mixed with multiple micronutrient powder. The soy flavor of YYB may explain that the taste of YYB was unacceptable by some children. A previous study showed that improved YYB which added peanut and sesame could be more acceptable [12].Furthermore, high mineral concentration in MNP sachets could also influence the taste. Therefore, careful attention must be given to the supplements’ sensory characteristics during the development process to minimize cases of rejection and to increase adherence to intervention [36].

Real–life program implementation challenges can be hard to predict. It is known that effectiveness of MNP depends on caregivers to be motivated to offer sachets MNP for children properly and without interruption [36]. Experience form Bangladesh MNP program also suggested that maintaining the supply chain of micronutrient powders was one of the key success factors to MNP program [37]. However, in our study YYB was stocked out for twice due to a period of over six months of the complex governmental approval process, which likely will have caused a significant decrease in adherence in the Mini 2 survey. Therefore, uninterrupted flow of MNP to the community has to be maintained in the future as well. Another challenge was that there was a continual increase in the number of caregivers who temporarily stopped giving YYB due to their forgetfulness in our study. The possible reason was that more than half caregivers expressed they did not perceive improvements in their children’s after taking YYB, and the proportion increased over time as well. It is documented that perceived benefits to children’s health was one of factors contributed to high acceptability among caregivers [36] and a visible and convincing change in nutrition status of children is another key success factor to MNP program [37]. Studies also indicated that a longer duration of the intervention decreased people’s motivations and adherence [31,35]. Additional efforts should be planned in reinforce caregivers’ knowledge on the benefits of YYB, such as text message reminder, which has been proved could improve the compliance of caregivers to a home fortification program [38]. Moreover, currently no governmental subsidies are in place to compensate village doctors’ time and cost and this is an obstacle to sustainability of the program.

### Strengths and limitations

A strength of this evaluation study is that we collected data from four follow–up cross-sectional surveys which showed the trends over time in program implementation. Our evaluation study also has several limitations. First, the main indicator “high adherence to YYB (children who consumed at least four YYB sachets during the previous week)” in this paper was based the caregivers’ recalled data during the previous week, which may have recall bias. Previous studies defined “high adherence to MNP” on weekly basis [6] that is “consumption of four sachets or more per week”; however, we could not get the data on weekly basis, the one week data could not completely represent the real consumption during the whole intervention period. Future monitoring could introduce a compliance card similar to an immunization card to keep track of children under the program [30]. Furthermore, this evaluation study took place in one Chinese county and caution is needed when generalizing the findings from this study to other settings. When similar evaluations in different settings are conducted, this data can be compared to our setting in China.

## CONCLUSIONS

Monitoring YYB distribution and consumption promoted the YYB program implementation, which could reveal issues affecting adherence to and acceptability of YYB, and direct more strategic implementation. Village doctors were critical to the success of the Chinese community–based YYB programs as they distribute the supplements and educate caregivers; quality training conducted among village doctors could improve the caregivers’ awareness of YYB, thus improve children adherence to YYB. Efforts to improve adherence in the community–based complementary food supplements include: improving the taste of the food supplement, strengthening health education of village doctors and caregivers, and ensuring continuity of food supplement supply. Future programs also need to monitor program implementation in other settings in China and elsewhere.
