# Supplementary material for: Monitoring and evaluating the adherence to a complementary food supplement (Ying Yang Bao) among young children in rural Qinghai, China: a mixed methods evaluation study
Source: J Glob Health. 2017 Jun 13;7(1):011101. doi: 10.7189/jogh.07.011101 (PMC5502707; doi:10.7189/jogh.07.011101)
Supplement: Online Supplementary Document [file jogh-07-011101-s001.pdf]

Online Supplementary Document

Wu et al. Monitoring and evaluating the adherence to a complementary food supplement (Ying Yang Bao) among young children in rural Qinghai, China: a mixed methods evaluation study

J Glob Health 2017;7:011101

Appendix S1. Questionnaire.

1. Identification（ID）

**ID.1a**	County：___________	**ID.1an**□□
**ID.1b**	Township：___________	**ID.1bn**□□
**ID.1c**	Village：___________	**ID.1cn**□□
**ID.2**	interviewee：___________	**ID.2a**□□
**ID.3**	The realtionship between the interviewee and the child：<br>1.Mother 2.father 3.Grandparets 8.others：______	**ID.3**□
**ID.4**	Main caregiver or not？ 1.Yes 2.No	**ID.4**□
**ID.5**	Child name _________________	**ID.5**
**ID.6**	Sex of the child：1.Boy 2.Girl	**ID.6**□
**ID.7**	Birthday of the child：<br>__ __ __ __year__ __month__ __day	**ID.7** □□□□/□□/□□
**ID.8**	Birth weight：__ __ __ __ g（8888.Don't konw）	**ID.8**□□□□
**ID.9**	Birth height：__ __.__cm （88.8.Don't konw）	**ID.9**□□. □
**ID.10**	How many children has mother given birth before this child？<br>__ （If≥7, then fill"7", 8. Don't konw）	**ID.10**□
**ID.11**	Was mother anemic during the pregancy?<br>1.Yes 2.No 8.Don't know	**ID.11**□
**ID.12**	What was the gestational age of the child？<br>__ __weeks（88.Don't konw）	**ID.12**□□
**ID.13**	What type of Hukou does the child have?<br>1.Urban 2.Rural	**ID.13**□
**ID.14**	Interviewer：___________	**ID.14**□□

**ID.15**	Survey date：__ __ __ __year__ __month__ __day	**ID.15**□□□□/□□/□□

2. Breastfeeding and Nutrition（BN）

**I now want to ask you about how young children should be fed. This refers to children younger than 2 years in general, not seocifically to your child.**		
**BN.1**	Can you tell me until what age a baby should receive only breastmilk, that is , no other food, water or teas？	**BN.1**☐☐
	__ __months [less than 1 month = 00，Don't know =88]	
**BN.2**	Call you tell me at what age a baby should start receiving foods such as porridge, mashed or solid foods?？	**BN.2**☐☐
	__ __months [less than 1 month = 00，Don't know =88]	
**BN.3**	Can you tell until what age a child should be breastfed？	**BN.3**☐☐
	__ __age in months [less than 1 month = 00]<br>90. When the milk dries out<br>91. when the child no longer wants the breast<br>88．Don' know	
**BN.4.**	Can you tell me until what age a child should be fed lean meat or liver?	**BN.4.f**☐☐
	__ __months， [less than 1 month = 00，Don't know =88]	

**Now I want to ask you about how your child is being fed.**		
**BN.5**	Has the child ever been breastfed?	**BN.5**☐
	1. Yes 2. No ——> ***BN.6a***	
**BN.6**	Was the child breastfed yesterday？	**BN.6**☐
	1. Yes——>__ __times in last 24 hours*[Don't know=88.]*<br>2. No, my child hasn't been weaning, but did not be breastfed yesterday.<br>3. No. My child has been weaning——>At what age did the child be weaning？__ __ 月*[Don't know=88.]*<br>8. Don't know	**BN.6.1**☐☐<br><br>**BN.6.3**☐☐

**BN.7**	**Next I would like to ask you about some liquids that the child may have had yesterday during the day or at night. Did the child have any (item from list)? Read the List of Liquids.'**	
Vitamin/medicine/liquid	During the last 24 hours？	
1. Vitamin/mineral supplements or drugs	1. Yes 2. No 8. Don't know	BN.7.1☐
2. ORS	1. Yes 2. No 8. Don't know	BN.7.2☐
3. Plain water/bottle water/suger water/tea	1. Yes 2. No 8. Don't know	BN.7.3☐
4. Infant formula	1. Yes___ __times *(88.* Don't know *)*<br>2. No 8. Don't know	BN.7.4☐<br>BN.7.4.1☐☐
5. Milk such as tinned, powdered, or fresh animal milk	1. Yes __ __times（*88.* Don't know）<br>2. No 8. Don't know	BN.7.5☐<br>BN.7.5.1☐☐
6. Juice or juice drinks	1. Yes 2. No 8. Don't know	BN.7.6☐
7. Clear broth	1. Yes 2. No 8. Don't know	BN.7.7☐
8. Yogurt	1. Yes __ __times（*88.* Don't know）<br>2. No 8. Don't know	BN.7.8☐<br>BN.7.8.1☐☐
9. Thin porridge	1. Yes 2. No 8. Don't know	BN.7.9☐
10. Lactic-acid drink	1. Yes 2. No 8. Don't know	BN.7.10☐
11. Others：______________	1. Yes 2. No 8. Don't know	BN.7.11☐

**BN.8**	Please describe everything that the child ate yesterday during the day or night, whether at home or outside the home.<br>Yesterday during the day or night, did your child drink/eat any (food group items)?		

**Food group items**	CodIng categorIes	
1. Porridge, bread, rice, noodles, or other foods made from grains	1.Yes 2.No 8.Don't know	BN.8.1☐
2.Pumpkin, carrots, squash, or sweet potatoes that are yellow or orange inside	1.Yes 2.No 8.Don't know	BN.8.2☐
3. White potatoes, white yams, manioc, cassava, or any other foods made from roots	1.Yes 2.No 8.Don't know	BN.8.3☐
4. Any dark green leafy vegetables	1.Yes 2.No 8.Don't know	BN.8.4☐
5. Ripe mangoes, ripe papayas, or (insert other local vitamin a-rich fruits)	1.Yes 2.No 8.Don't know	BN.8.5☐
6. Any other fruits or vegetables	1.Yes 2.No 8.Don't know	BN.8.6☐
7. Liver, kidney, heart, or other organ meats	1.Yes 2.No 8.Don't know	BN.8.7☐
8. any lean meat, such as beef, pork, lamb, goat, chicken, or duck	1.Yes 2.No 8.Don't know	BN.8.8☐
9. Any fat meat	1.Yes 2.No 8.Don't know	BN.8.9☐
10. Eggs	1.Yes 2.No 8.Don't know	BN.8.10☐
11. Fresh or dried fish, shellfish, or seafood	1.Yes 2.No 8.Don't know	BN.8.11☐
12. Any foods made from beans, peas, lentils, nuts, or seeds	1.Yes 2.No 8.Don't know	BN.8.12☐
13. Cheese, yogurt, or other milk products	1.Yes 2.No 8.Don't know	BN.8.13☐
14. Any oil, fats, or butter, or foods made with any of these	1.Yes 2.No 8.Don't know	BN.8.14☐
15.Any sugary foods such as chocolates, sweets, candies, pastries, cakes, or biscuits	1.Yes 2.No 8.Don't know	BN.8.15☐
16.Condiments for flavor, such as chilies, spices, herbs, or fish powder	1.Yes 2.No 8.Don't know	BN.8.16☐
17. Sausage	1.Yes 2.No 8.Don't know	BN.8.17☐
**Others:**		

**BN.9**	Did the child eat any solid, semi-solid, or soft foods yesterday during the day or at night?	**BN.9**☐☐
	1. Yes，At what aged the child started eating __ __months<br>（Less than one month = 00，88.Don' t know/remember）<br>2. No——> **BN.12a**	**BN.9.1**☐☐
**BN.10**	How many times did the child eat solid, semisolid, or soft foods other than liquids yesterday during the day or at night?	**BN.10**☐
	___ times *[If≥7，fill "7"；8=Don't know]*	

Now I would like to ask you about some particular foods (NAME) may eat. I am interested in whether your child had the item even if it was combined with other foods.

**BN.12.a**	Yesterday, during the day or night, did (NAME) consume any [list iron fortified solid, semisolid or soft foods designed specifically for infants and young children available in the local setting]?	**BN.12.a**☐
	1. Yes 2. No 8. Don't know	
**BN.12.b**	Yesterday, during the day or night, did (NAME) consume any [list lipid based nutrient supplement (lns) available in the local setting]?	**BN.12.b**☐
	1. Yes 2. No 8. Don't know	

3. YingYangBao（YYB）

**YYB.1**	Have you ever received YYB？	**YYB.1□**
	1. Yes ——>***YYB.2***<br>2. No<br>8. Don't know——>***YYB.12***	
**YYB.1a**	Why didn't you receive YYB？ ——>***YYB.12***	**YYB.1.a□**
	1. Dno't want<br>2. Not at home when distribution<br>3. Didn't know YYB distribution<br>4. This were no YYB left in the village clinics<br>5. Others,specify_________________________________<br>8. Don't know	
**YYB.2**	How old was your child when you started receiving YYB? __ __months （88.Don't know）	**YYB.2□□**
**YYB.3**	Until now, how many boxes of YYB have you ever received since you started receiving YYB? __ __ boxes（30sachets/box）？ **（"Less than one box", fill"00", 88.Don't know）**	**YYB.3□□**
**YYB.4**	Has your child ever conusmed YYB？	**YYB.4□**
	1. Yes——>***YYB.5***<br>2. No<br>8. Don't know——>***YYB.12***	
**YYB.4a**	Why didn't you feed your child YYB？ ——>***YYB.12***	**YYB.4a□**
	1. My child dislike taking<br>2. busy and have no time to feed<br>3. YYB was useless, and did not want to feed to my child<br>4. Didn't think YYB is a good thing as it was free<br>5. Adverse affects after taking, such as dierrhea<br>6. My child was too young to eat<br>7. Others,specify_________________________________<br>8. Don't know	
**YYB.2.a**	How old was your child when he/she started consuming YYB? __ __months（88.Don't know）	**YYB.2.a□□**
**YYB.13**	Until now, how many boxes of YYB have you ever consumed since you started receiving YYB? __ __ boxes（30sachets/box）？ **（"Less than one box", fill"00", 88.Don't know）**	**YYB.13□□**
**YYB.5.a**	Does your chid stop consuming YYB now?	**YYB.5.a□□**
	1.No——>***YYB.5.c***<br>2.Yes<br>8.Don't know——>***YYB.12***	
**YYB.5.b**	Why did your child stop consuming YYB？ ——>***YYB.12***	**YYB.5.b□□**
	1. My child dislike taking<br>2. busy and have no time to feed<br>3. YYB was useless, and did not want to feed to my child<br>4. Didn't think YYB is a good thing as it was free<br>5. Adverse affects after taking, such as dierrhea	

	6. The taste of YYB was not good<br>7. Others,specify____________________________________<br>8. Don't know	
**YYB.5.c**	Did your child consume YYB yesterday?	**YYB.5.c**□
	1.Yes 2. No 8. Don't konw	
**YYB.5.d**	How many sachets of YYB did your child consumed during the previous week?”<br>__ __sachets （**"Less than one sachets", fill"00", 88.Don't know**）	**YYB.5.d**□□
**YYB.6**	In which situations you won't feed YYB to your child?（**Mutiple-choice**）	
	1. Diarrhea 1. Refer 2. Not refer	**YYB.6.1**□
	2. Got cold 1. Refer 2. Not refer	**YYB.6.2**□
	3. Forget 1. Refer 2. Not refer	**YYB.6.3**□
	4. Suspect adverse affects 1. Refer 2. Not refer	**YYB.6.4**□
	5. Don't konw wether YYB is good or bad to my child 1. Refer 2. Not refer	**YYB.6.5**□
	6. Others, specify_____________ 1. Refer 2. Not refer	**YYB.6.6**□
	7. My child dislike consuming 1. Refer 2. Not refer	**YYB.6.7**□
	8 Don't know 1. Refer 2. Not refer	**YYB.6.8**□
**YYB.7**	How do you think your child like taking YYB?	**YYB.7**□
	1. Like very much<br>2. Liked<br>3. Neutral<br>4. Disliked at the beginning, but liked after a while<br>5. Disliked, reasons for dislike ____<br>8. Don’t know.	
**YYB.8**	Do you think YYB is a meal which can play as complementary food?	**YYB.8**
	1. Yes<br>2. No<br>8. Don't know	
**YYB.9**	What changes do you think your child have after taking YYB?（**Mutiple-choice**）	
	1. No changes observed 1. Refer 2. Not refer	**YYB.9.1**□
	2. Positive weight gains 1. Refer 2. Not refer	**YYB.9.2**□
	3. Positive height gains 1. Refer 2. Not refer	**YYB.9.3**□
	4. Increased appetite 1. Refer 2. Not refer	**YYB.9.4**□
	5. Prevented diseases 1. Refer 2. Not refer	**YYB.9.5**□
	6. Increased cognitive ability, more smart 1. Refer 2. Not refer	**YYB.9.6**□
	7. Others, specify____________________ 1. Refer 2. Not refer	**YYB.9.7**□
	8. Don't know 1. Refer 2. Not refer	**YYB.9.8**□
**YYB.10**	Did your child have any adverse reaction when he/she start taking YYB?	**YYB.10**□
	1. Yes， specify____<br>2. No<br>8 Don't know	
**YYB.11**	Would you insist feeding YYB to your child everyday?	**YYB.11**□
	1. Yes——>***YYB.12***<br>2. NO<br>8. Don't know——>***YYB.12***	
**YYB.11a**	Why wouldn't you be able to feed your child YYB everyday?	**YYB.11a**□

	1. My child dislike taking<br>2. Forget<br>3. There is other things at home<br>4. Busy<br>5. Didn't think YYB is a good thing as it was free<br>6. YYB is no effect<br>7. Did not reveive<br>8. Don't know<br>9. Others, specify____		
**YYB.12**	Have you ever received infamation on YYB, such as what is YYB, how and Why to feed YYB to your child?		**YYB.12**□
	1. Yes<br>2. No<br>8 Don't konw		
**YYB.12a**	Where did you receive information on YYB?（**Mutiple-choice**）		
	1.Information on YYB boxes	1. Refer 2. No refer	**YYB.12a.1**□
	2.the leaflets which called "A letter to caregivers"	1. Refer 2. No refer	**YYB.12a.2**□
	3.Village doctors	1. Refer 2. No refer	**YYB.12a.3**□
	4.TV	1. Refer 2. No refer	**YYB.12a.4**□
	5.Banners	1. Refer 2. No refer	**YYB.12a.5**□
	6.Neighor	1. Refer 2. No refer	**YYB.12a.6**□
	7.Others, specify ____________________	1. Refer 2. No refer	**YYB.12a.7**□
	8.Don't know	1. Refer 2. No refer	**YYB.12a.8**□
	9.Short text message	1. Refer 2. No refer	**YYB.12a.9**□

4. Cough and fever（CO）

**CO.1**	Has the child been ill with a fever at any time in the last two weeks?		**CO.1** □
	1. Yes 2. No 8. Don't know		
**CO.2**	Has the child had an illness with a cough at any tiem in the last two weeks?		**CO.2** □
	1. Yes<br>2. NO——> ***CO.3***<br>8. Don't know——> ***CO.3***		
**CO.2.a**	When the child had an illness with a cough, did he/she breathe faster than usual with short, quick breaths or have diffivulty breathing?		**CO.2.a**□
	1. Yes<br>2. No——>***CO.3 check***<br>8. Don't know——>***CO.3 check***		
**CO.2.b**	Wew the symptoms due to a problem in the chest or a blocked nose？		**CO.2.b**□
	1. Chest<br>2. Blocked nose<br>3. Both<br>4. Others：______________________________<br>8. Don't know		
**CO.3**	**<u>Check</u>：CO.1 or CO.2**<br>**1. There is "1.yes" answer in CO.1 or CO.2 ——> CO.4;**<br>**2. There is no "1.yes" in both CO.1 and CO.2——>*DI.1***		**CO.3**□
**CO.4**	Did you seek advice or treatment for the fever/cough outside home?		**CO.4**□
	1. Yes<br>2. No——>***CO.5***<br>8. Don't know——>***CO.5***		
**CO.4.a**	Where did you seek advice or treatment？ **[*Record all sources mentioned。Prompt*** *"Anywehere esle？"* **]**		
	**1. Family member/ friend**<br>11 Famliy member<br>12 Friends/ Neighbour	 1. Yes 2. No<br>1. Yes 2. No	 **CO. 4.a.11**□<br>**CO. 4.a.12**□
	**2. Public Health Sector**<br>21 Hospital of county or above<br>22 MCH hospital<br>23 Communtity health center<br>24 Township hospital<br>25 Communtity health station<br>26Village clinic	 1. Yes 2. No<br>1. Yes 2. No<br>1. Yes 2. No<br>1. Yes 2. No<br>1. Yes 2. No<br>1. Yes 2. No	

	**3. Private Health Sector**<br>31 Hospital<br>32 Private clinic<br>33 Pharmacy	 1. Yes 2. No<br>1. Yes 2. No<br>1. Yes 2. No	
	**4．Community**<br>41 Brith attendant<br>42 Family planing staff	 1.Yes 2. No<br>1. Yes 2. No	
	**51. Others**，specify___________________________	1. Yes 2. No	
**CO.5**	Wa the child given medicine for fever or cough？		**CO.5**□
	1. Yes<br>2. No<br>8. Don’t know		
**CO.6**	How did it last from onset to recover fully? __ __ Day （If≥87, fill“87”, Don’t know =88， Not recover=90）		**CO.6**□□

5. Diarrhea（DI）

**DI.1**	Has the child had diarrhea in the last 2 weeks?		**DI.1**□
	1. Yes<br>2. No ——>***HH.1***<br>8. Don’t know ——>***HH.1***		
**DI.2**	Did the child have blood in the stools?		DI.2□
	1. Yes<br>2. No<br>8. Don’t know		
**DI.3**	During this last episode of diarrhea, did the child drink any of the following： ***（Read each item aloudand record response before proceeding to the next）***		
	1. ORS	1.Yes 2. No 8.don’t know	**DI.3.1**□
	2. ORT（Plain water/bottle water、porridge）	1.Yes 2. No 8.don’t know	**DI.3.2**□
	3.Other homemade fluids（eg，Tea、soft drink）____________	1.Yes 2. No 8.don’t know	**DI.3.3**□
**DI.3a**	During the child illness, did he/she drink much less, about the sam, or more than usual?<br>(If less, porbe: Was he/she offered much less than usual to drink or somewhat less?)		**DI.3a**□
	1. Much less or none<br>2. Somewhat less<br>3. About the same<br>4. More<br>8. Don’t know		
**DI.3b**	When the child had diarrhea, did he/she eat less, about the same, or more food than usual?<br>(If less, porbe: much less or a little less?)		**DI.3b**□
	1.Much less or none<br>2. Somewhat les<br>3. About the same<br>4. More<br>5. Child never received solid or semi-solid foods<br>8. Don’t know		
**DI.4**	Did you seek advice or treatment for the diarrhea outside home?		**DI.4**□
	1. Yes<br>2. No——>***DI.5***<br>8. Don’t know——>***DI.5***		
**DI.4.a**	Where did you seek advice or treatment? **[*Record all sources mentioned。Prompt*** *“Anywehere esle？”* ***]***		
	**1. Family member/ friend**		
	11 Famliy member	1. Yes 2. No	**DI.4.a.11**□
	12 Friends/ Neighbour	1. Yes 2. No	**DI.4.a.12**□

	**2. Public Health Sector**		
	21 Hospital of county or above	1. Yes 2. No	**DI._4.a.21**☐
	22 MCH hospital	1. Yes 2. No	**DI. 4.a.22**☐
	23 Communtity health center	1. Yes 2. No	**DI. 4.a.23**☐
	24 Township hospital	1. Yes 2. No	**DI. 4.a.24**☐
	25 Communtity health station	1. Yes 2. No	**DI. 4.a.25**☐
	26Village clinic	1. Yes 2. No	**DI. 4.a.26**☐
	**3. Private Health Sector**		
	31 Hospital	1. Yes 2. No	**DI. 4.a.31**☐
	32 Private clinic	1. Yes 2. No	**DI. 4.a.32**☐
	33 Pharmacy	1. Yes 2. No	**DI. 4.a.33**☐
	**4. Community**		
	41 Brith attendant	1.Yes 2. No	**DI. 4.a.41**☐
	42 Family planing staff	1. Yes 2. No	**DI. 4.a.42**☐
	**51. Others**，specify________________________	1. Yes 2. No	**DI. 4.a.51**☐
**DI.5**	How did it last from onset to recover fully? __ __ Day （If≥87，fill“87”，Don’t know =88， Not recover=90）		**DI.5**☐☐

6. Household information

**HH.1**	Household size？ __ __	**HH.1**□□
**HH.2**	How many adults (>16 years) in your family? __	**HH.2**□
**HH.3**	How many under five children in your family? __	**HH.3**□
**HH.4**	Age of the mother：__ __years	**HH.4**□□
**HH.5**	Enthic of the mohter<br>1. Han<br>2. Hui<br>3. Tu<br>4. Tibetan<br>5. Sala<br>6. Mongolia<br>7. Others _____________	**HH.5**□
**HH.6**	Education of the mother?<br>1.Never go to school<br>2.Primary school<br>3.Midddle school<br>4.High school<br>5. technical secondary school<br>6. Junior collge<br>7. Collge or above<br>8. Don’t know	**HH.6**□
**HH.7**	How long has the mother been at school？<br>__ __ years （00.Never，88. Don’t know）	**HH.7**□□
**HH.8**	Mother’s work<br>01.Household work<br>02. Manager in enterprise or<br>03. Professionals<br>04. General clerk<br>05. Business services personne<br>06. individual business<br>07. Engaged in non-agricultural labor of farmers<br>08. worker<br>09. famers<br>10. Soldier<br>11.Other____________________<br>88.Don’t know	**HH.8**□□
**HH.9**	During the previous year, did the mother work outside home/hometown?<br>1. Yes<br>2. No——>***HH10a***	**HH.9**□
**HH.9a**	How old was the child when his/her mother go out for worker?__ __months（**”less than one month” fill “00”. “Don’t know” fill “88”**）	**HH.9a**□□
**HH.9b**	How long did the mother work outside work during the previous year：__ __ months（**”less than one month” fill “00”. “Don’t know” fill “88”**）	**HH.9b**□□
**HH.10**	Age of the father：__ __years	**HH.10**□□
**HH.11**	Enthic of the fahter<br>1. Han<br>2. Hui<br>3. Tu<br>4. Tibetan<br>5. Sala<br>6. Mongolia<br>7. Others _____________	**HH.11**□
**HH.12**	Education of the father?<br>1.Never go to school<br>2.Primary school<br>3.Midddle school<br>4.High school<br>5. technical secondary school<br>6. Junior collge<br>7. Collge or above<br>8. Don’t know	**HH.12**□
**HH.12a**	How long has the father been at school？	**HH.12a**□□

	__ __ year （00.Never，88. Don’t know）		
**HH.13**	Father’s work		**HH.13**□□
	01.Household work<br>02. Manager in enterprise or<br>03. Professionals<br>04. General clerk<br>05. Business services personne<br>06. individual business	07. Engaged in non-agricultural labor of farmers<br>08. worker<br>09. famers<br>10. Soldier<br>11.Other____________________<br>88.Don’t know	
**HH.14**	During the previous year, did the father work outside home/hometown?		**HH.14**□
	1. Yes<br>2. No——>***HH10a***		
**HH.14a**	How old was the child when his/her father go out for worker?__ __months（**”less than one month” fill “00”. “Don’t know” fill “88”**）		**HH.14a**□□
**HH.14b**	How long did the father work outside work during the previous year：__ __ months（**”less than one month” fill “00”. “Don’t know” fill “88”**）		**HH.14b**□□
**HH.16**	Who is the main caregivers of the child？		**HH.16**□
	1. Mother——>***HH.17***<br>2. Father——>***HH.17***<br>3. Grandparents<br>4. Others______________________		
**HH.16a**	Age of the main caregiver of the child：__ __years		**HH.16a**□□
**HH.16b**	Education of the main caregiver?		**HH.16b**□
	1.Never go to school<br>2.Primary school<br>3.Midddle school<br>4.High school	5. technical secondary school<br>6. Junior collge<br>7. Collge or above<br>8. Don’t know	
**HH.16c**	How long has the main caregiver been at school？		**HH.16c**□□
	__ __ year （00.Never，88. Don’t know）		

**HH.16d**	Father’s work		**HH.16d**□□
	01.Household work<br>02. Manager in enterprise or<br>03. Professionals<br>04. General clerk<br>05. Business services personne<br>06. individual business	07. Engaged in non-agricultural labor of farmers<br>08. worker<br>09. famers<br>10. Soldier<br>11.Other____________________<br>88.Don’t know	
**HH.17**	Main income source of family？		**HH.17**□
	1. Grain planting<br>2. Vegetable planting<br>3. Animal Husbandry<br>4. forestry	5. Business<br>6. Work for others<br>7. Others ________________________<br>8. Don’t know	
